# A nomogram combining clinical variables and MR imaging features for predicting response in head-neck cancer

**DOI:** 10.1186/s13244-025-02196-y

**Published:** 2026-01-27

**Authors:** Xinyan Wang, Yiming Ding, Hangzhi Liu, Changyu Zhu, Xiaoxia Qu, Yue Kang, Cong Ding, Yuchen Wang, Meiling Mao, Zhinxin Li, Xiaohong Chen, Junfang Xian

**Affiliations:** 1 Department of Radiology, Beijing Tongren Hospital, Capital Medical University, Beijing, China; 2 Department of Otolaryngology Head and Neck Surgery, Beijing Tongren Hospital, Capital Medical University, Beijing, China; 3 Cancer Center, Beijing Tongren Hospital, Capital Medical University, Beijing, China; 4 Department of Pathology, China-Japan Friendship Hospital, Beijing, China

**Keywords:** Head and neck squamous cell carcinoma, Neoadjuvant therapy, Chemoimmunotherapy, Treatment efficacy prediction, Multiparametric magnetic resonance imaging

## Abstract

**Objectives:**

This study aims to develop a multimodal nomogram to predict neoadjuvant chemoimmunotherapy (NCIT) outcomes in head and neck squamous cell carcinoma (HNSCC).

**Materials and methods:**

Treatment-naive HNSCC patients receiving neoadjuvant NCIT were retrospectively analyzed. Clinical information, conventional MR imaging features, dynamic contrast-enhanced-MRI (DCE-MRI) parameters and ADC values were analyzed in relation to pathological complete response (pCR). The predictive accuracy of clinical and MRI parameters was evaluated using the receiver operating characteristic (ROC) curve, with the area under the curve (AUC) serving as a key metric.

**Results:**

Following NCIT, 55.0% (67/122) of patients achieved pCR. Significant differences were observed in clinical variables, including tumor location, combined positive score (CPS) and neutrophil-to-lymphocyte ratio (NLR) between pCR and non-pCR groups (*p* < 0.05). Imaging features (tumor margin, growth pattern, T2 homogeneity, necrosis, three distinct enhancement patterns, tumor diameter and lymph node short-axis diameter) also differed significantly (*p* < 0.05). The enhancement pattern was the most efficient predictor of pCR (AUC = 0.83). A combined model incorporating CPS, tumor diameter, and enhancement pattern achieved an AUC of 0.86. The baseline K^trans^ and ADC values demonstrated an AUC of 0.712 and 0.715 for pCR prediction. The H&E-stained whole-slide analyses revealed significant correlations between specific MRI features and tumor lymphocyte densities/ratios.

**Conclusions:**

We developed a novel combined model integrating CPS and routine pretreatment MRI features to predict NCIT response in HNSCC. The enhancement pattern was the strongest predictor of pCR, while functional MRI parameters also showed significant predictive value.

**Critical relevance statement:**

This study demonstrates that systematically integrating combined positive score with routine pretreatment MRI features can effectively predict neoadjuvant chemoimmunotherapy response. These findings may help optimize therapeutic strategies for head and neck squamous cell carcinoma.

**Key Points:**

- Predicting neoadjuvant chemoimmunotherapy response in head and neck cancer remains challenging.
- A novel clinical-MRI model improves chemoimmunotherapy response prediction in head-neck cancer.
- The three enhancement patterns emerged as the most robust predictors.

**Graphical Abstract:**

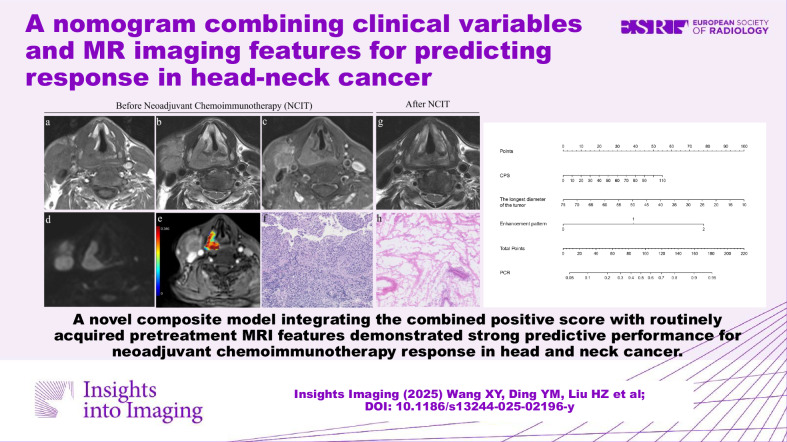

## Introduction

Head and neck squamous cell carcinoma (HNSCC) is a prevalent malignancy with significant morbidity and mortality worldwide, ranking as the sixth most common cancer worldwide [1]. Despite advances in conventional therapies, the prognosis for advanced, recurrent or metastatic HNSCC remains poor, with a 5-year survival rate of approximately 40–53% [2]. The introduction of immunotherapy, particularly immune checkpoint inhibitors, has revolutionized the treatment landscape and influenced the prognosis of HNSCC [3]. Clinical trials have demonstrated that immune checkpoint inhibitors with or without chemotherapy can significantly improve overall survival and progression-free survival in locally advanced and recurrent or metastatic HNSCC [4, 5].

However, assessing treatment response to neoadjuvant immunotherapy and chemoimmunotherapy remains a significant challenge [6]. The RECIST criteria frequently fall short in accurately predicting pathological response, as they primarily focus on tumor size reduction. This approach does not fully consider immunotherapy-induced changes, like lymphocyte infiltration into the tumor microenvironment, which may result in pseudoprogression [7]. Previous studies have reported misclassification of disease progression in up to 30% of cases using RECIST criteria [8]. Commonly used biomarkers, such as the combined positive score (CPS)—defined as the sum of all PDL1–positive cells (tumor cells, lymphocytes, and macrophages) divided by the total number of viable tumor cells, then multiplied by 100—require invasive biopsies and exhibit limited accuracy when used as standalone predictors of treatment response [9]. Given these challenges and the high cost of immunotherapy, there is an urgent need to develop comprehensive predictive models that integrate multiple parameters for accurate, non-invasive treatment response assessment.

MRI is the preferred imaging technique for the diagnosis and evaluation of HNSCC, as conventional imaging features can provide clinically actionable insights without requiring complex computational analysis. Multiparametric MRI integrates conventional MRI features with advanced techniques, offering the potential to address significant challenges in evaluating responses to immunotherapy [10]. Several recent studies have demonstrated the potential of MRI, including diffusion-weighted imaging (DWI) and dynamic contrast-enhanced MRI (DCE-MRI), in predicting immunotherapy response in HNSCC [11–14]. However, these studies are limited by small sample sizes and have primarily focused on individual MRI modalities, lacking a comprehensive investigation that integrates multiple MRI techniques and clinical factors. Currently, there is no integrated predictive model that combines clinical parameters (e.g., CPS score) with pretreatment MRI features to improve assessment accuracy.

Therefore, this study aims to explore the potential of multiparametric MRI, in conjunction with clinical factors, to enhance the predictive accuracy of treatment response in HNSCC, ultimately contributing to more personalized and effective therapeutic approaches.

## Materials and methods

### Study patients

**Fig. 1:**
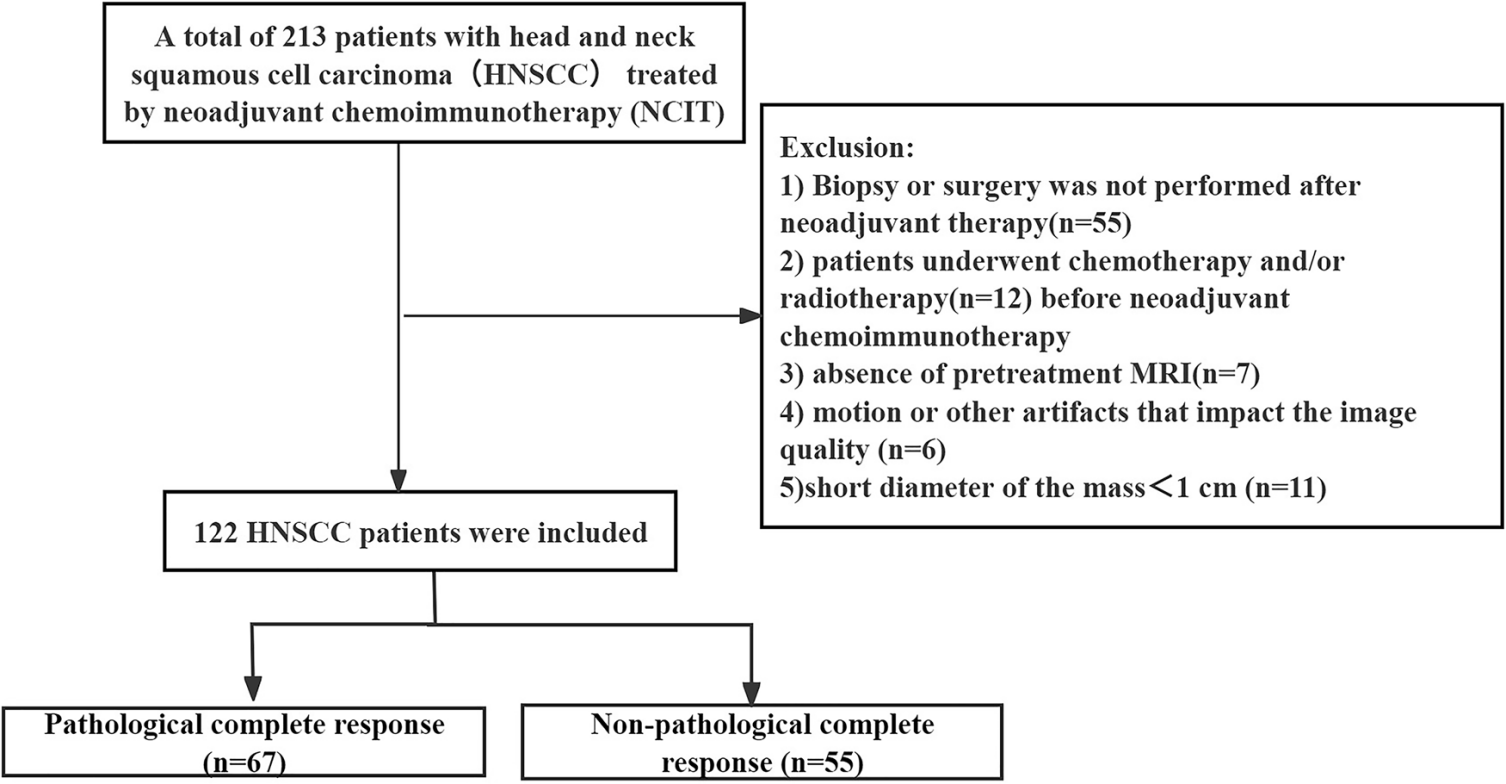
Flowchart of the study sample

This retrospective investigation received approval from the Ethics Committee of the Beijing Tongren Hospital (TREC02023-KY011), and the requirement for written informed consent was waived. From July 2021 to July 2024, a total of 213 consecutive HNSCC patients treated by NCIT in our institution from a prospective clinical trial were screened (TREC2023-KY011). The exclusion criteria included absence of pretreatment contrast-enhanced T1-weighted MRI, patients who underwent radiotherapy or chemotherapy before neoadjuvant, poor MRI quality and short diameter of the mass < 1 cm. Finally, 122 HNSCC patients were included in the analysis (Fig. 1). The patients received NCIT 2–3 cycles with cisplatin (75–80 mg/m² body surface area (BSA)) on days 1–3 and paclitaxel liposome (135–150 mg/m² BSA) on day 1. The anti-PDL1 pembrolizumab (200 mg, day 1) or tislelizumab (200 mg, day 1) was administered on day 5.

Baseline clinical information, including age, sex, HPV status, combined positive score (CPS), neutrophil-to-lymphocyte ratio (NLR), and other relevant factors, was obtained from the patient.

### Image acquisition

MRI examinations were conducted in 60 patients with a 3-T scanner (MAGNETOM Prisma, Siemens Healthcare) using a 64-channel head and neck coil and in 62 patients with a GE HDxt 3.0-T MR scanner (GE Healthcare) using a 20-channel head and neck coil. All patients underwent pre-contrast axial T1WI and T2WI as well as post-contrast fast spin echo (FSE) T1WI in axial, coronal, and sagittal views. DCE-MRI and DWI were performed in 60 patients on the 3-T Prisma scanner.

The parameters for the 3-T Prisma scanner and the GE HDxt 3.0-T MRI system are provided in Supplementary Document 1 (S1).

### Imaging analysis

**Fig. 2:**
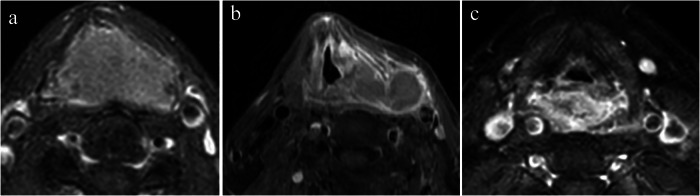
Examples for T1-weighted contrast enhancement patterns. Homogeneous enhancement is defined as a uniform and even distribution of contrast material on contrast-enhanced T1-weighted MR images, with minimal heterogeneity or necrosis (**a**). Homogeneous enhancement with an outer ring sign (**b**) is characterized by a uniformly enhanced central region (homogeneous enhancement) surrounded by a well-defined, hyperintense peripheral rim (outer ring sign). Heterogeneous enhancement (**c**) is characterized by a non-uniform distribution of signal intensity on contrast-enhanced T1-weighted MR images

Two board-certified head and neck radiologists (X.W. (16 years’ experience) and H.L. (6 years’ experience)) independently interpreted all imaging studies while blinded to pathological outcomes and treatment responses. Discordant interpretations were resolved through consensus review with a third senior neuroradiologist (J.X., 32 years’ experience) using standardized criteria. Conventional MR imaging features including tumor location (hypopharynx, oropharynx, larynx or others), margin, tumor growth pattern (infiltrative or mass-forming), T2 signal intensity, T2 homogeneity, T1-weighted contrast enhancement pattern (homogeneous enhancement, homogeneous enhancement with an outer ring sign or heterogeneous enhancement) (Fig. 2), necrosis of the tumor, the longest and shortest diameter of the tumor, lymph node necrosis, lymph node margin, and lymph node short-axis diameter were analyzed. Homogeneous enhancement was defined as a uniform and even distribution of contrast material on contrast-enhanced T1-weighted MR images, with minimal heterogeneity or necrosis. Homogeneous enhancement with an outer ring sign was characterized by a uniformly enhanced central region (homogeneous enhancement) surrounded by a well-defined, hyperintense peripheral rim (outer ring sign).

The DWI and DCE-MRI data were processed using Siemens Advantage Workstation (MR DCE V1.1.2). A small ROI (about 30 mm²) with the lowest ADC value was drawn on the b = 0 image to calculate ADC. For DCE-MRI analysis, an individual arterial input function (AIF) was generated by placing an ROI in the internal carotid artery 1 cm above the bifurcation on the lesion side. The DCE parametric metrics (K^trans^, K_ep_ and V_e_) were calculated using the modified two-compartment Tofts model. The whole slice ROIs were manually delineated on the slice with the strongest enhancement, excluding necrotic, vascular, cystic, and hemorrhagic areas.

### Pathological response evaluation

After completion of two or three rounds of NCIT, all patients proceeded to undergo surgical or endoscopic procedures involving multiple re-biopsies to assess the pathological response. The treatment outcome was evaluated based on whether a pathological complete response (pCR) or non-pathological complete response (non-pCR) was achieved. A pCR refers to the absence of residual invasive cancer cells in the tumor bed. Biopsies that exhibited fibrosis, hemorrhage, or significant chronic and active inflammation were interpreted as indicative of the prior tumor location.

### Model interpretability

Biopsy samples prior to induction immunochemotherapy for a subset of patients were obtained. The H&E-stained whole-slide tissue scan analyses were conducted to investigate how imaging features influenced the predictive model for response to neoadjuvant chemoimmunotherapy in HNSCC. The cell detection and classification process was facilitated by the QuPath open-source software platform. For each tissue slice, six random fields measuring 250 × 250 μm were selected for analysis. Tumor-infiltrating lymphocytes (TILs) demonstrated prognostic significance in HNSCC. Through dual-pathologist evaluation, tumor-region cells were classified into four categories: tumor cells, lymphocytes, fibroblasts, and other components (Supplementary Fig. 1). We quantified: (1) cellular densities (total, tumor, lymphocyte, fibroblast) and (2) lymphocyte ratios, including stromal (esTILs%), total (etTILs%), and enhanced (eTILs%) TIL percentages:

1. esTILs% = 100 × (lymphocytes density / stromal cell density). Stromal cell density = total cell density − tumor cell density.
2. etTILs% = 100 × (lymphocytes density / total cell density).
3. eTILs% = 100 × (lymphocytes density / (lymphocytes + tumor cell density)).

### Statistical analysis

To assess the differences in conventional MRI features, ADC values, and DCE-MRI metrics between the pCR and non-pCR groups, chi-square tests or Mann–Whitney U tests were employed. Variables that exhibited statistically significant differences (*p* < 0.05) in univariate analysis were included in a multivariate logistic regression model. To determine the optimal thresholds, receiver operating characteristic (ROC) curve analysis and area under the curve (AUC) calculations were performed for significant variables. The thresholds were selected to maximize the sum of sensitivity and specificity. Sensitivity, specificity, accuracy, and areas under the ROC curves (AUCs) for response prediction were calculated based on established cut-off values. AUC comparisons were conducted using DeLong’s test. All statistical analyses were performed using SPSS version 25.0 (SPSS Inc.) and MedCalc software (version 19.6).

## Results

### Patient characteristics

The demographic and tumor characteristics of the included participants are summarized in Table 1. A total of 122 HNSCC patients (median age, 59 years; range, 30–77 years) were included in the analysis. Of these, only one patient (0.82%) was female, while the remaining 121 (99.18%) were male. Hypopharyngeal squamous cell carcinoma was the most prevalent subtype (68/122, 55.7%), followed by oropharyngeal squamous cell carcinoma, which accounted for 27 cases (22.1%). A significant difference in AJCC staging was observed between various primary sites, with a higher prevalence of stage II and III tumors in laryngeal squamous cell carcinoma and oropharyngeal squamous cell carcinoma (*p* < 0.001).

**Table 1:** Patients’ baseline clinicopathological characteristics

Characteristic	All patients (*n* = 122)	Patients with pCR (*n* = 67)	Patients without pCR (*n* = 55)	*p*-value
Age (years)				0.61
Median age, years (range)	59 (30–77)	60 (43–77)	58 (30–76)	
Gender				0.45
Female	1	0	1	
Male	121	67	54	
Location				**0.007**
Hypopharynx	68	36	32	
Oropharynx	27	16	11	
Larynx	19	13	6	
Others	8	2	6	
Histologic differentiation				0.27
Poor	50	23	27	
Well-moderate	49	28	21	
Unknown	23	16	7	
HPV status				0.30
Positive	19	12	7	
Negative	92	49	43	
Unknown	11	6	5	
CPS (median, range)	20 (1–101)	25 (1–101)	10 (1–100)	**0.006**
NLR				**0.028**
< 4.5	103	61	42	
> 4.5	19	6	13	
T stage				0.20
T1–T3	72	43	29	
T4	50	24	26	
N stage				0.19
N0–N1	50	31	19	
N2–N3	72	36	36	
AJCC stage 8th edition				
Ⅱ	14	10	4	0.19
III	29	18	11	0.38
Ⅳa	72	36	36	0.10
Ⅳb	4	3	1	
Ⅳc	3	0	3	

Boldface values indicate statistically significant results, defined as *p*-values less than 0.05

### Comparison of clinicopathologic characteristics according to pathologic response

Following NCIT, 67 of the 122 patients with HNSCC (55.0%) achieved a pathological complete response (pCR). Significant differences in tumor location, CPS and NLR values were observed between the pCR and non-pCR groups (*p* = 0.007, *p* = 0.006, and *p* = 0.028, respectively) (Table 1). The pCR rate was highest in laryngeal squamous cell carcinoma, at 13 out of 19 (68.4%), followed by oropharyngeal squamous cell carcinoma, with a pCR rate of 16 out of 27 (59.3%). CPS values in the pCR group were significantly higher compared to those in the non-pCR group (*p* = 0.006). In the pCR group (*n* = 67), only 6 patients (9.0%) had NLR values above 4.5, compared with 13 patients (23.6%) in the non-pCR group (*p* = 0.027).

There was no association between baseline characteristics and pCR in terms of age, gender, histopathological type, HPV status, T stage, N stage and clinical stage (Table 1).

### Comparison of baseline conventional MRI parameters according to pathologic response

**Fig. 3:**
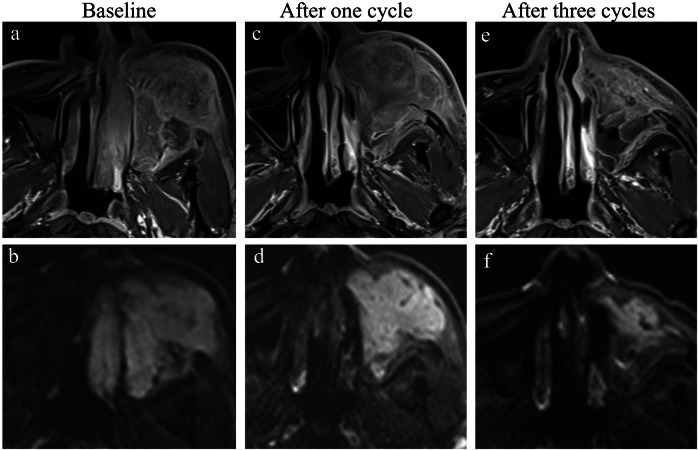
MRI findings in a patient with poorly differentiated squamous cell carcinoma in the sinonasal region, demonstrating non-pathologic complete response after three cycles of neoadjuvant chemoimmunotherapy (NCIT). **a** Baseline contrast-enhanced T1-weighted image (CET1WI) and (**b**) diffusion-weighted image (DWI) demonstrate a lesion located in the left nasal cavity and maxillary sinus region. The lesion in the nasal cavity shows a homogeneous enhancement pattern, while the lesion in the maxillary sinus region exhibits markedly heterogeneous enhancement. Follow-up CET1WI (**c**) and DWI (**d**) after one cycle of NCIT reveals complete resolution of the nasal cavity lesion, with a large residual lesion persisting in the left maxillary sinus region. **e**, **f** After three cycles of NCIT, the nasal cavity lesion remains absent, while the residual lesion in the maxillary sinus region is still present

**Fig. 4:**
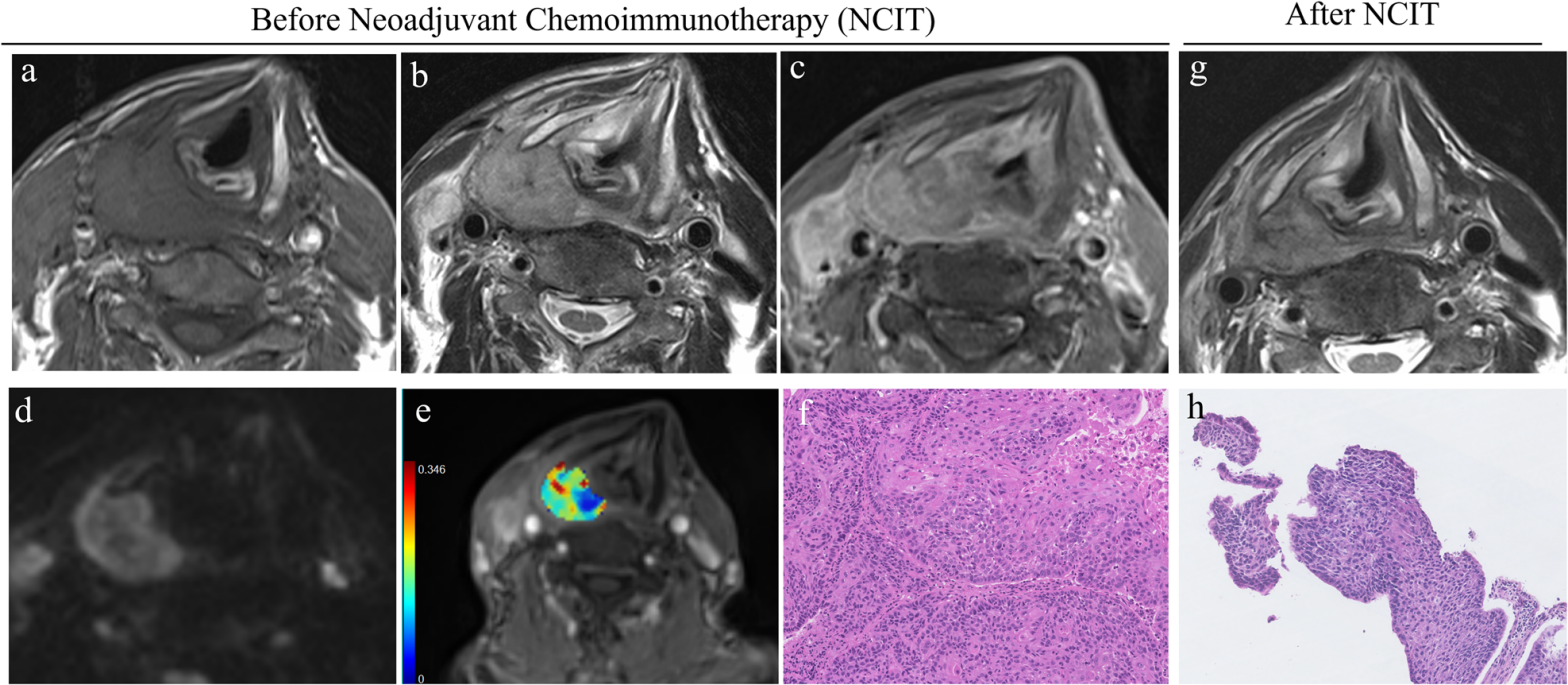
MRI and histopathological findings in a patient with stage Ⅳb hypopharyngeal carcinoma exhibiting a non-pathological complete response. Axial T1-weighted image (**a**) and T2-weighted image (**b**) show a lesion in the right hypopharynx involving the right thyroid cartilage plate. **c** Post-contrast T1-weighted image reveals marked heterogeneous enhancement of the lesion, accompanied by right cervical lymph node metastasis and extracapsular extension. **d** Diffusion-weighted imaging (DWI) shows diffusion restriction within the lesion. **e** K^trans^ map derived from dynamic contrast-enhanced MRI indicates a K^trans^ value of 0.28/min, reflecting moderate vascular permeability. **f** Hematoxylin and eosin (H&E) staining (× 200 magnification) of the pathological section demonstrates a heterogeneous mixture of tumor cells and stromal components, with scattered inflammatory cell infiltration. **g** T2-weighted image after three cycles of treatment shows partial reduction in lesion size, with residual tumor still present. **h** Post-treatment H&E staining (× 200 magnification) reveals residual viable tumor cells and scattered inflammatory cell infiltration, consistent with non-pathological complete response (non-pCR)

**Fig. 5:**
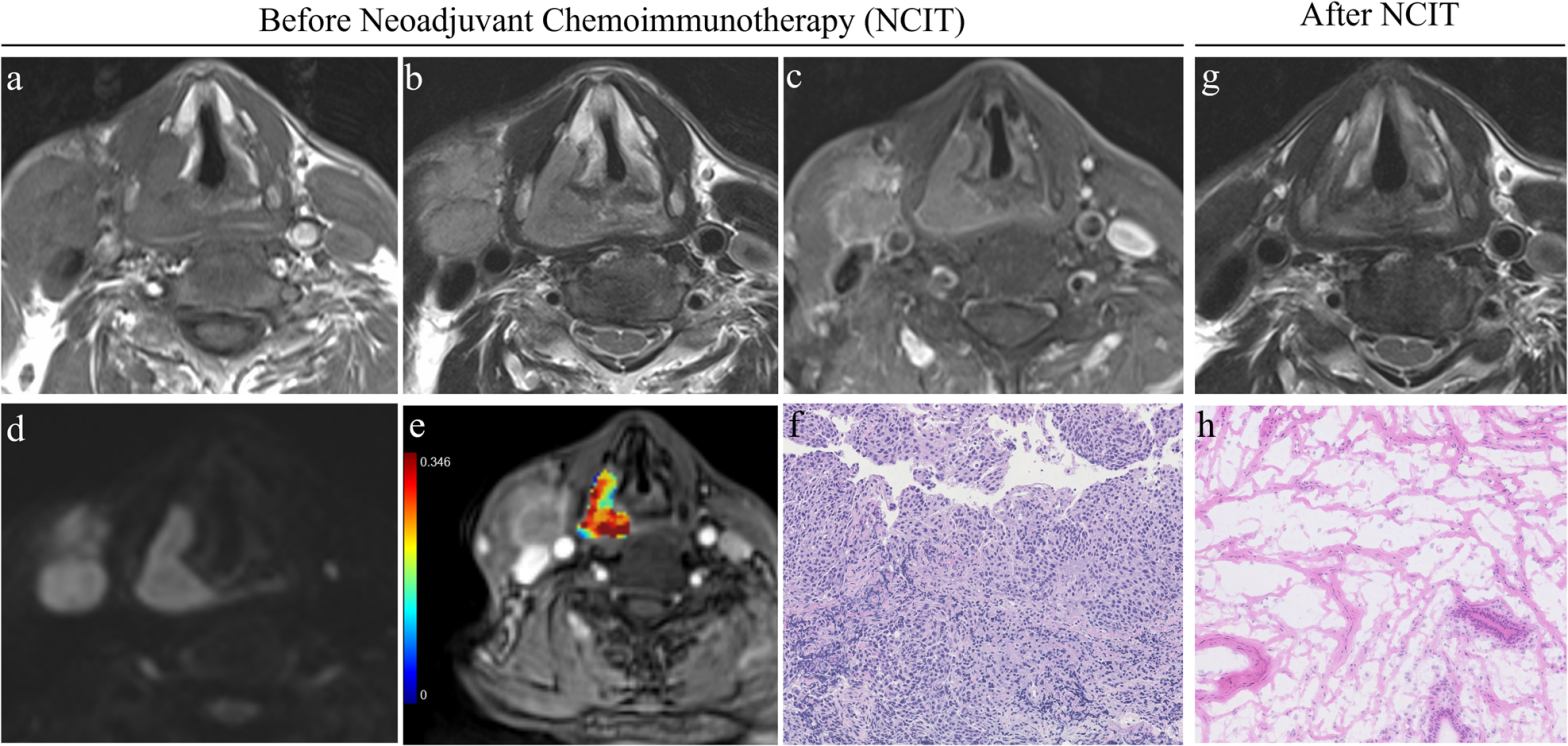
MRI and histopathological findings in a patient with stage Ⅳb hypopharyngeal carcinoma achieving pathological complete response (pCR). Axial T1-weighted image (**a**) and T2-weighted image (**b**) show a lesion in the right hypopharynx, with homogenous signal intensity. Post-contrast T1-weighted image (**c**) reveals homogeneous enhancement of the lesion, accompanied by right cervical lymph node metastasis and extracapsular extension. **d** Diffusion-weighted imaging (DWI) shows marked diffusion restriction within the lesion. **e** K^trans^ map derived from dynamic contrast-enhanced MRI indicates a K^trans^ value of 0.35/min, reflecting marked vascular permeability. **f** Hematoxylin and eosin (H&E) staining (× 200 magnification) of the pathological section demonstrates tumor cells with prominent inflammatory cell infiltration and minimal stromal components. **g** T2-weighted image after three cycles of treatment shows no obvious residual tumor detected. **h** Post-treatment H&E staining (× 200 magnification) reveals extensive fibrosis, scattered inflammatory cell infiltration, and no viable tumor cells, consistent with pathological complete response (pCR)

Significant differences were found in tumor margin, tumor growth pattern, T2 homogeneity, tumor necrosis, enhancement pattern, tumor diameter and lymph node diameter (Table 2). A homogeneous enhancement pattern was more frequently observed in the pCR group (45/67, 67.2%), while a heterogeneous pattern predominated in the non-pCR group (43/55, 78.2%) (Figs. 3–5). The frequency of homogeneous enhancement with an outer ring sign showed no significant difference between the two groups (*p* = 0.51). Ill-defined margins, infiltrative tumor growth pattern and heterogeneous T2 signal intensity were more frequently observed in the non-pCR group.

**Table 2:** Patients’ conventional MRI parameters according to pathologic response

	Patients with pCR		Patients without pCR		*p*-value
	No.	%	No.	%	
No. of patients	67	100	55	100	
Tumor margin					**0.045**
Well-defined	39	58	22	40	
Ill-defined	28	42	33	60	
Tumor growth pattern					**0.03**
Infiltrative	31	46	36	65	
Mass-forming	36	54	19	35	
T2 signal intensity					0.23
Hypointense	0	0	1	2	
Isointense	38	57	25	45	
Hyperintense	29	43	29	53	
T2 homogeneity					**< 0.001**
Homogenous	48	72	14	25	
Heterogeneous	19	28	41	75	
Enhancement pattern					**< 0.001**
Homogeneous	45	67	6	11	
Heterogeneous	12	18	43	78	
Homogeneous enhancement with an outer ring sign	10	15	6	11	
Tumor necrosis					**< 0.001**
Yes	12	18	30	55	
No	55	82	25	45	
Lymph node necrosis					
Yes	41	61	36	65	0.65
No	9	13	10	18	
Lymph node margin					0.79
Well-defined	14	21	14	25	
Ill-defined	36	54	32	58	
The longest diameter of the tumor (mm) (range)	28 (13–55)		34 (19–74)		**< 0.001**
The shortest diameter of the tumor (mm) (range)	17 (10–48)		23 (6–51)		**0.001**
Short-axis diameter of the lymph node (mm)	17 (8–46)		13 (6–42)		**0.008**

*pCR* pathological complete response

Boldface values indicate statistically significant results, defined as *p*-values less than 0.05

### Predictive performance of clinical factors and conventional MRI parameters

**Fig. 6:**
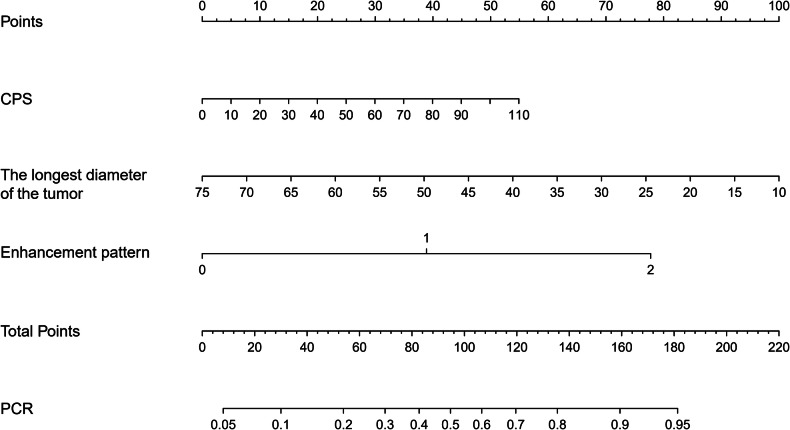
Clinical-MRI nomogram for the prediction of pathologic complete response to neoadjuvant chemoimmunotherapy in head and neck squamous cell carcinoma

In predicting pCR status, the most efficient feature was the enhancement pattern, which achieved an AUC of 0.83 (95% CI: 0.75–0.91), with a sensitivity of 82% and specificity of 78% (Table 3). By integrating CPS, the longest tumor diameter and enhancement pattern in the combined model, an AUC of 0.86 (95% CI: 0.78–0.93) was achieved (Fig. 6). The combined model demonstrated an overall accuracy of 82.1%, sensitivity of 84.6%, specificity of 78.7%, positive predictive value (PPV) of 84.6%, and negative predictive value (NPV) of 78.7%. A marginal difference was observed between the combined model and the enhancement pattern for predicting pCR (*p* = 0.059).

**Table 3:** Performance of clinical factors and MRI parameters for predicting pCR status

Variable or model	AUC	Accuracy (%)	Sensitivity (%)	Specificity (%)	PPV (%)	NPV (%)
CPS	0.65	64	71	55	69	58
NLR	0.55	61	92	22	59	71
Tumor margin	0.59	59	58	60	64	54
Tumor growth pattern	0.60	59	54	66	65	54
T2 homogeneity	0.73	73	72	75	77	68
Enhancement pattern	0.83	80	82	78	82	78
Tumor necrosis	0.68	70	82	55	69	71
LD of the tumor (mm)	0.70	64	79	45	64	64
SD of the tumor (mm)	0.67	62	81	40	62	63
SD of the lymph node (mm)	0.66	59	56	63	62	57
Combined model	0.86	82	85	79	85	79

*CPS* combined positive score, *NLR* neutrophil-to-lymphocyte ratio, *LD* longest diameter, *SD* shortest diameter, *pCR* pathological complete response, *PPV* positive predictive value, *NPV* negative predictive value

### Comparison of functional MRI parameters according to pathologic response

The baseline K^trans^ and K_ep_ values in the pCR group were significantly higher than those in the non-pCR group, while baseline ADC values were significantly lower than those in the non-pCR group (*p* < 0.001, *p* = 0.03 and *p* = 0.004, respectively) (Table 4). The baseline K^trans^ value demonstrated an AUC of 0.712 (95% CI: 0.56–0.85) for pCR, with an accuracy of 70%, sensitivity of 79%, specificity of 59%, PPV of 70%, and NPV of 70%. Similarly, the ADC value achieved an AUC of 0.715 (95% CI: 0.58–0.85), with an accuracy of 72%, sensitivity of 64%, specificity of 82%, PPV of 81%, and NPV of 65% (Supplementary Fig. 2). No significant difference was observed between the two parameters in predicting pCR (*p* = 0.97). When baseline K^trans^ and ADC values were combined in a model, the resulting AUC was 0.731. No statistically significant differences were observed between the combined model and the individual models of K^trans^ (*p* = 0.72) and ADC (*p* = 0.83).

**Table 4:** The comparisons of DCE-MRI quantitative parameters and ADC according to pathologic response

Functional MRI parameters median (25%, 75% quartile)	Patients with pCR	Patients without pCR	*p*-value
Baseline K^trans^ (/min)	0.12 (0.07, 0.14)	0.07 (0.05, 0.09)	**< 0.001**
Baseline V_e_	0.28 (0.16, 0.42)	0.21 (0.12, 0.27)	0.20
Baseline K_ep_ (/min)	0.62 (0.50, 1.00)	0.56 (0.28, 0.63)	**0.03**
Baseline ADC (× 10^−3^mm^2^/s)	1.00 (0.91, 1.13)	1.21 (1.08, 1.61)	**0.004**

*pCR* pathological complete response

Boldface values indicate statistically significant results, defined as *p*-values less than 0.05

### Correlations between imaging features and pathologic characteristics

The H&E-stained whole-slide tissue scan analyses were conducted in 25 patients with HNSCC. The results revealed that some MRI imaging features were associated with lymphocyte densities and lymphocyte ratios. The patients with homogeneous enhancement patterns showed higher lymphocytes densities ((1190.11 ± 814.73)/mm^2^ vs (299.41 ± 304.38)/mm^2^, *p* < 0.001), etTILs% (11.62% ± 9.85% vs 5.00% ± 5.62%, *p* = 0.006) and eTILs% (18.50% ± 14.37% vs 7.34% ± 9.85%, *p* = 0.005) than the patients with heterogeneous enhancement patterns. Among patients with different T2 homogeneity, marginal differences were observed in the percentages of etTILs (*p* = 0.06) and eTILs (*p* = 0.07). No significant differences were observed in the pathologic characteristics among various tumor margin and necrosis patterns (*p* > 0.05).

## Discussion

Given the challenges in treatment response evaluation and the substantial costs of neoadjuvant chemoimmunotherapy, we developed a clinically practical nomogram integrating established predictive markers. Our optimized model combines clinical parameters (CPS) with readily assessable MRI features, including enhancement patterns and tumor diameter. The enhancement pattern emerged as the strongest individual predictor (AUC = 0.83), while the integrated nomogram demonstrated superior performance (AUC = 0.86), thereby providing a practical tool for predicting treatment response. The quantitative MRI parameters (K^trans^, K_ep_ and ADC values) showed clinical promise in preliminary analyses; however, their formal inclusion was constrained by the limited number of patients who underwent DCE and DWI on the same scanner within our cohort.

Tumor location was found to be an important clinical factor for predicting pCR in our study. The pCR rate of laryngeal cancer (13/19, 68%) and oropharyngeal squamous cell carcinoma (16/27, 59%) was higher in this study. Previous literature has indicated that the early symptoms of laryngeal and oropharyngeal cancer often lead to an early-stage tumor diagnosis at the initial visit, resulting in a relatively good treatment outcome [15]. Our findings support this observation, demonstrating a pathologic complete response (pCR) rate of 71.4% (20/28) in stage II-III patients, compared to 50% (9/18) in stage IV laryngeal and oropharyngeal cancers (*p* = 0.142), reinforcing the association between earlier-stage diagnosis and improved treatment outcomes. However, this difference did not reach statistical significance, necessitating further validation with a larger sample size to confirm these findings.

The combined positive score (CPS), integrating the expression of programmed death-ligand 1 (PDL1) on both tumor and immune cells, demonstrated significant differences in our study. This finding is consistent with previous studies [16], which have suggested that a higher CPS score may be predictive of a more favorable treatment response in HNSCC and other cancer types. NLR, as a comprehensive indicator of inflammation and immune response, reflects the systemic immune status of patients. Previous studies have also shown that a high NLR is associated with a poor prognosis [17, 18], which is further verified in our study. We observed a higher number of patients with NLR greater than 4.5 in the non-pCR group.

In terms of imaging, conventional MRI features such as tumor margin, tumor growth pattern, T2 homogeneity, tumor necrosis, enhancement pattern and tumor diameter showed significant differences between the pCR group and the non-pCR group. Specifically, the pCR group more often presented with homogeneous enhancement patterns, while the non-pCR group more frequently showed heterogeneous enhancement patterns. Although radiomics studies have demonstrated a correlation between tumor heterogeneity and therapeutic efficacy [19, 20], the clinical translation of these methods remains a work in progress. In this study, we aim to explore the potential of visually assessed tumor heterogeneity as a unique and readily accessible biomarker for predicting treatment response, leveraging its immediate applicability in clinical practice. The observed correlation between homogeneous enhancement and higher pathological complete response (pCR) rates may be explained by increased intratumoral lymphocyte infiltration and decreased stromal content in homogeneously enhancing tumors—a finding supported by our preliminary data. Our future work will systematically investigate the associations between three distinct enhancement patterns (homogeneous enhancement, homogeneous enhancement with an outer ring sign, and heterogeneous enhancement) and tumor immune microenvironment subtypes (immune-inflamed, immune-excluded, and immune-desert phenotypes). The derived parameter K^trans^ from DCE-MRI enhances the evaluation of therapeutic outcomes by providing quantitative information on tumor microenvironmental characteristics, serving as a critical adjunct to conventional MRI for evaluating treatment efficacy [21]. A higher pretreatment K^trans^ value is associated with improved therapeutic efficacy, which has been validated in head and neck squamous cell carcinoma (HNSCC) as well as other types of tumors [13, 22]. Our study showed that higher K^trans^ values in pCR patients with head and neck carcinomas respond more effectively to treatment, with sensitivity and specificity of 79% and 59%, respectively (AUC = 0.712). The observed association between higher K^trans^ values and improved therapeutic efficacy may be explained by the underlying vascular characteristics of these tumors. Elevated K^trans^ values indicate a more permeable vascular network, and the increased vascular permeability enhances the delivery of immunochemotherapy drugs. Additionally, the results of our study demonstrate that HNSCC with lower ADC values are associated with better therapeutic efficacy and a higher pCR rate. Although the relationship between ADC values and immunotherapy response in HNSCC remains less established and shows inconsistency in the literature [23], our findings align with the established paradigm that low-ADC tumors are more susceptible to chemotherapy, leading to higher pCR rates [24]. We propose that the low baseline ADC observed in the pCR group reflects a tumor microenvironment characterized by high cellular density and a reduced stromal component, which may lead to decreased interstitial fluid pressure, thereby enhancing drug perfusion and penetration and ultimately contributing to improved therapeutic outcomes [24]. Furthermore, changes in ADC values between pre- and post-treatment are considered a more reliable predictor of therapeutic efficacy [25], and this will be a focus of our subsequent investigations.

It should be noted that although this study has made some valuable discoveries in predicting the response to neoadjuvant chemoimmunotherapy, there are still some limitations. First, the sample size is relatively small, which may limit the generalizability of the results. Second, this study is a single-center study, and the results may be affected by center-specific factors. Therefore, future multicenter, large-sample studies are needed to further validate our findings. Third, this study is limited by incomplete functional MRI data, as only 60 out of 122 patients with HNSCC underwent both DCE-MRI and DW-MRI. This precluded a comprehensive analysis integrating conventional MRI features with functional MRI parameters for predicting therapeutic efficacy, potentially limiting the depth of our predictive modeling. Future studies should aim for more complete functional imaging datasets to enhance the robustness of such multimodal analyses. In conclusion, this study has developed the first clinical-MRI nomogram integrating CPS with routinely available pretreatment imaging features to predict NCIT response in HNSCC. The model utilizes standard-of-care MRI parameters (enhancement patterns and tumor diameters) without requiring advanced post-processing, enabling immediate implementation in most oncology centers. Future multicenter studies should validate its utility for personalizing locoregional and systemic therapy strategies in the immunotherapy era.

## Supplementary information

Supplementary Material

## Data Availability

Data are available on reasonable request. The datasets are available from the corresponding authors on reasonable request.

## Abbreviations

ADC: Apparent diffusion coefficient
AUC: Area under the curve
CPS: Combined positive score
DCE-MRI: Dynamic contrast-enhanced-MRI
DWI: Diffusion-weighted imaging
HNSCC: Head and neck squamous cell carcinoma
NCIT: Neoadjuvant chemoimmunotherapy
NLR: Neutrophil-to-lymphocyte ratio
NPV: Negative predictive value
pCR: Pathological complete response
PPV: Positive predictive value
ROC: Receiver operating characteristic curve
TIL: Tumor-infiltrating lymphocytes

## Declarations

### Cohort overlap

Twenty (20) patients included in this current study were also part of a previously published cohort (Liu et al [14]). However, the two studies are distinct in their methodologies and conclusions. The previous study focused on predicting pathological complete response (pCR) to neoadjuvant chemo-immunotherapy using MRI histogram analysis.
