# Supplementary material for: A nomogram combining clinical variables and MR imaging features for predicting response in head-neck cancer
Source: Insights Imaging. 2026 Jan 27;17:25. doi: 10.1186/s13244-025-02196-y (PMC12847587; doi:10.1186/s13244-025-02196-y)
Supplement: Supplementary file 1 — Supplementary Material [file 13244_2025_2196_MOESM1_ESM.pdf]

A Nomogram Combining Clinical Variables and MR Imaging Features for Predicting Response in Head-Neck Cancer

ELECTRONIC SUPPLEMENTARY MATERIAL

The parameters on the 3T Prisma scanner were as follows: turbo spin echo (TSE) T1WI: TR 420 ~ 440ms, TE 9-12ms; TSE T2WI: TR 4500 ~ 4820ms, TE 90 ~ 102ms; matrix 352 × 228. The parameters on the GE HDxt 3.0T MRI system were as follows: fast spin echo (FSE) T1WI (TR 600 ~ 700ms, TE 8ms), FSE T2WI (TR 3900 ~ 4300ms, TE 90ms), matrix 320 × 224. Parameters on the above two scanners: field of view 22 × 22 cm; slice thickness 5.5mm; gap 1 mm.

DW-MRI was performed in 60 patients by using RESOLVE-DWI (readout segmentation of long variable echo-trains diffusion-weighted imaging) on the Prisma 3.0T MR scanner. Parameters were as follows: b-values of 0 and 1000s/mm^2, TR 2950ms, TE 52-84ms, 16 sections, slice thickness 5.5 mm, 1.1 mm intersection gap, field of view of 24×24 cm and matrix of 140×140.

DCE-MRI was completed in 60 patients by using ultrafast TWIST-VIBE sequence (time-resolved angiography with stochastic trajectories and volume-interpolated breath-hold examination) the Prisma 3.0T MR scanner: TR 4.2ms, TE1=1.33 ms, TE2=2.56 ms, flip angle（FA）9°, slice thickness 5mm and matrix 352 × 249. A total of 35 consecutive scans with a temporal resolution of 1.54 seconds were acquired. Gadopentetate dimeglumine (Magnevist; Bayer Schering, Berlin, Germany) was administered intravenously at a dose of 0.1 mmol/kg, with an injection rate of 2 mL/s, followed by a 20-mL saline flush.

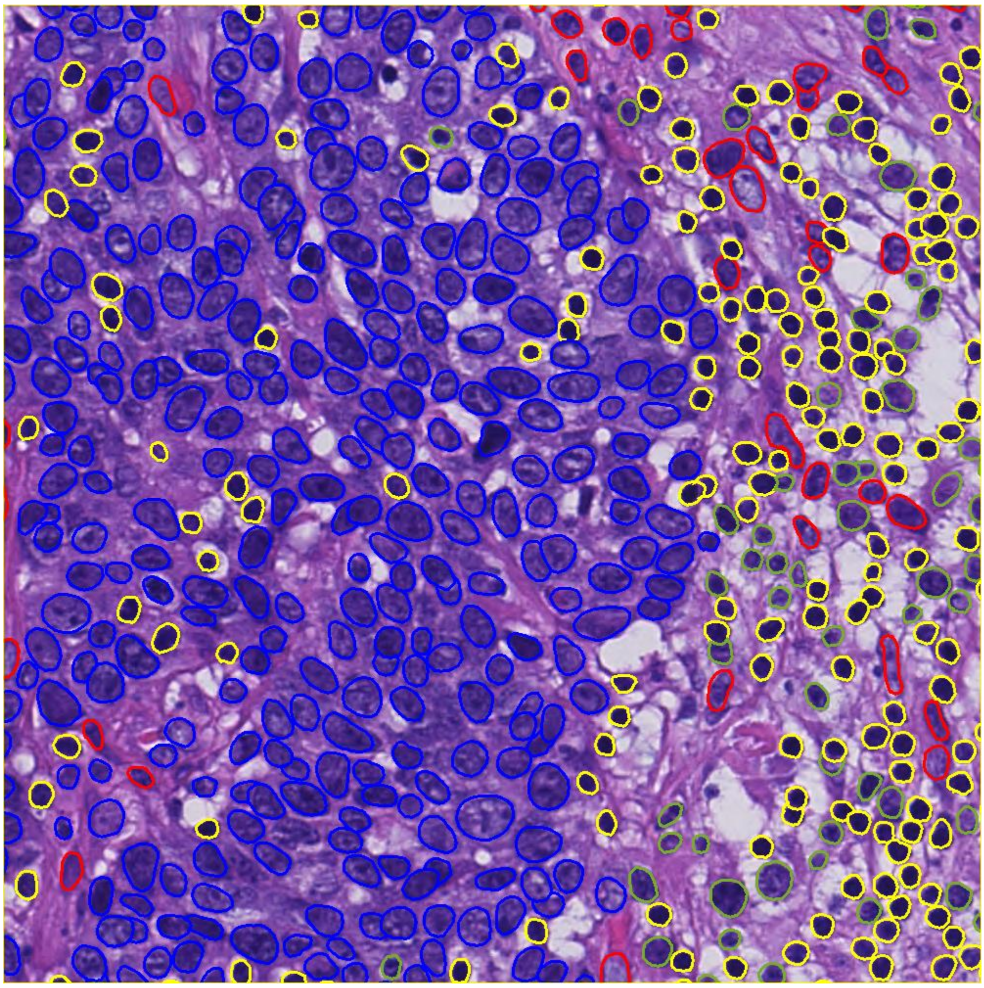

Figure S1 Cells detected and classified into four categories

Cells identified within six randomly selected fields, each measuring 250 microns by 250 microns in whole-slide images, were classified into four distinct groups: tumor cells (blue), lymphocytes (yellow), fibroblasts (red), and other detections (green).

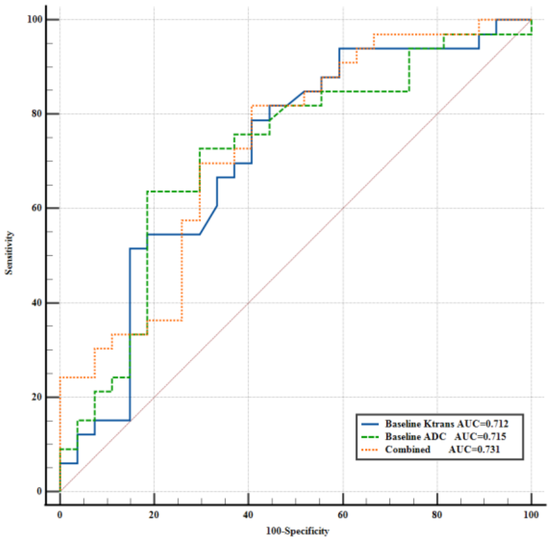

Fig. S2 Receiver operating characteristic curves for predicting treatment response to neoadjuvant chemoimmunotherapy. Comparisons of predictive performance of baseline K^{trans}, ADC and combined K^{trans} and ADC model revealed no statistically significant differences between the combined model and the individual models of K^{trans} (p = 0.718) and ADC (p = 0.833), respectively.
